# Measurement, Prediction, and Control of Individual Heart Rate Responses to Exercise—Basics and Options for Wearable Devices

**DOI:** 10.3389/fphys.2018.00778

**Published:** 2018-06-25

**Authors:** Melanie Ludwig, Katrin Hoffmann, Stefan Endler, Alexander Asteroth, Josef Wiemeyer

**Affiliations:** ^1^Department of Computer Sciences, Institute of Technology, Resource and Energy-Efficient Engineering, Bonn-Rhein-Sieg University of Applied Sciences, St. Augustin, Germany; ^2^Department of Human Sciences, Institute of Sport Science, Technical University of Darmstadt, Darmstadt, Germany; ^3^Department of Computer Science in Sports, Institute of Computer Science, Johannes Gutenberg University of Mainz, Mainz, Germany

**Keywords:** wearable sensors, heart rate modeling, heart rate control, heart rate prediction, phenomenological approaches, training monitoring, load control

## Abstract

The use of wearable devices or “wearables” in the physical activity domain has been increasing in the last years. These devices are used as training tools providing the user with detailed information about individual physiological responses and feedback to the physical training process. Advantages in sensor technology, miniaturization, energy consumption and processing power increased the usability of these wearables. Furthermore, available sensor technologies must be reliable, valid, and usable. Considering the variety of the existing sensors not all of them are suitable to be integrated in wearables. The application and development of wearables has to consider the characteristics of the physical training process to improve the effectiveness and efficiency as training tools. During physical training, it is essential to elicit individual optimal strain to evoke the desired adjustments to training. One important goal is to neither overstrain nor under challenge the user. Many wearables use heart rate as indicator for this individual strain. However, due to a variety of internal and external influencing factors, heart rate kinetics are highly variable making it difficult to control the stress eliciting individually optimal strain. For optimal training control it is essential to model and predict individual responses and adapt the external stress if necessary. Basis for this modeling is the valid and reliable recording of these individual responses. Depending on the heart rate kinetics and the obtained physiological data, different models and techniques are available that can be used for strain or training control. Aim of this review is to give an overview of measurement, prediction, and control of individual heart rate responses. Therefore, available sensor technologies measuring the individual heart rate responses are analyzed and approaches to model and predict these individual responses discussed. Additionally, the feasibility for wearables is analyzed.

## 1. Introduction

The use of wearable devices (“wearables”) as tools for training or activity tracking has increased considerably. More precise and accurate data acquisition due to improved sensor technology, advanced usability, and portability due to miniaturization and more powerful data analysis due to increased processing power allows the industry to introduce new and improved wearables (Chan et al., 2012; Mukhopadhyay, 2015). Therefore, wearables can be used as “every day” devices providing the user with detailed and individual information about physical activity (PA), fitness level, and physiological responses. Especially for non-athletes, wearables are claimed to be effective and efficient tools for physical training. “Find your own Fit” (Fitbit.com), “beat yesterday” (garmin.com), “listen to your body” (POLAR), or “Eat. Sleep. Move. Better” (Jawbone) are some of the slogans of well-known distributors of those wearables. In this context, especially the heart rate (HR) has become an often used indicator for individual cardiovascular strain during training. Exercise according to defined HR zones is already well established in professional and recreational endurance training. Several wearable devices do not only measure a person's heart rate, but might even give visual, acoustic, or vibro-tactile feedback if HR is outside a specified area. Most apps and devices are connected to web portals that provide a visualization of a subject's training data as well as more or less detailed recommendations for training.

The wide-spread use of HR is not surprising since the pumping action of the human heart is the driving force of blood circulation of the cardiovascular system. The main tasks of this system are to supply the cells with oxygen and nutrients, to remove carbon dioxide and metabolites, and to transport hormones, vitamins, and enzymes (Weiss and Jelkmann, 1989). This is especially apparent in the physical training process, when a defined external stimulus (i.e., load, pedal rate, velocity) is applied to the human body. The increased energy demand of the working muscles causes an increase in cardiovascular functions. Depending on the extent of individual strain (e.g., sleep or activity conditions) the heart has to sensitively adjust the ejection of blood to fulfill different demands of the human body. In contrast to other indicators of cardiovascular strain (e.g., stroke volume (SV), oxygen uptake (VO_2_), release of carbon dioxide (VCO_2_), metabolites as lactate or urea, and hormones) HR can be recorded non-invasive, with minimal technical effort, and without the constraints of laboratory conditions.

However, HR responds individually to physical stress or training load. Due to a high amount of internal (i.e., training status, genetics, mood) and external (i.e., environmental conditions, nutrition, water supply) influencing factors, the HR response can even fluctuate in the same individual during a single training session (Bunc et al., 1988; Ewing et al., 1991; Boushel et al., 2001; Achten and Jeukendrup, 2003; Bouchard and Hoffman, 2011; Hoffmann et al., 2016). By recording every single heartbeat, a high variation of longer and shorter heart cycles can be observed. This heart rate variability (HRV) is to a large extent modulated by the stimulating sympathetic and repressing parasympathetic influences of the Autonomous Nervous System (ANS) (Lacey, 1956; Stauss, 2003). Integrated in a variety of complexly nested regulatory mechanisms and reflexes, the antagonistic influences of ANS are modulated according to afferences from sensors that are situated throughout the human body. These sensors measure, e.g., changes in blood pressure, blood volume, or partial pressure of CO_2_ or O_2_ in the blood.

To evoke training responses corresponding to defined training goals, it is necessary to elicit individual optimal cardiovascular responses to neither overstrain nor under challenge the training person. Therefore, it is essential to model and predict these individual responses. This is the prerequisite for effective and efficient training.

Although the complex influence of reflexes and mechanisms on heart performance has been studied for centuries (e.g., Starling, 1918; Brandfonbrener et al., 1955), modeling and predicting every single heartbeat is yet not possible. In particular, the unpredictability of HRV must be considered as a source of error in modeling.

Therefore, the following HR kinetics need to be considered for modeling acute responses to stress:

- Short-term responses, expressed by the kinetics of HR to the onset or offset of load,
- Individual relationship of stress parameters and cardiopulmonary indicators.

This review aims at giving an overview of measurement, prediction, and control of individual HR responses. Therefore, different sensor technologies measuring HR and their feasibility for wearables are analyzed. Afterwards, current models of acute, individual HR responses are addressed, and the implementation and use cases of these models are discussed.

## 2. Measuring cardiac output via HR

HR kinetics can provide valuable information about the individual responses and therefore the individual strain of the human body. However, valid and reliable measurement of HR is essential to convey the required information and to enable a valid modeling and prediction of these responses. The following chapter analyzes the reliability of different sensor technologies currently available. Additionally, their feasibility for wearables is discussed.

The exclusive measurement of HR as a body's physiological response to exercise is widely used in several areas and applications. For example, HR is used to estimate a person's exhaustion or degree of fatigue (Vautier et al., 1994; She et al., 2013), to indicate individual cardiovascular function (Carter et al., 2003; Borresen and Lambert, 2008), to monitor exercise parameters (e.g., condition, intensity, exercise duration) of single persons or whole groups (Sornanathan and Khalil, 2010; Lee et al., 2015), or to control the individual training (Weghorn, 2013; Hunt and Hunt, 2016).

Due to the central location of the heart inside the torso and the vulnerability of the cardio-respiratory system, heart functions are often measured indirectly by acquiring signals that are caused by these functions. One possibility to measure cardiac output is by assessing SV. Although available measurement technologies (i.e., echocardiography, thermodilution, or direct Fick-method Smyth et al., 1984) show high reliability and validity and provide detailed information about the individual performance of the heart, none of them is suitable to be used during physical training. All described methods and techniques require a clinical setting and preferably a stationary participant.

An alternative way to measure cardiac output is by registering the individual HR or the electric and mechanical effects caused by the heartbeat. Due to the technological progress, new sensors and technologies for reliable and valid measurement of HR are available. Additionally, the sensors available so far still improve in quality and feasibility and allow for a more exact representation of the HR signal. At present, the following measuring technologies are used (see Table 1):

- Electrographic sensor
- Optical sensors
- Infrasonic cardiac vibration sensors
- Magnetic induction monitoring sensors
- Phonocardiographic sensors
- Sphygmographic sensors

**Table 1:** Feasibility of measurement techniques used in wearables.

**Sensor**	**Method**	**Author**	**Validity *r***	**Error sources**	**Feasibility for wearables**
Electrographic	ECG		1.00	• Electromagnetic waves	−
				• Motion artifacts	
				• Incorrect placement of electrodes	
	HR breast belt	Weippert et al., 2010	0.85–0.99	• Electromagnetic waves	+
				• Motion artifacts	
				• Disturbed signal conduction	
				• Incorrect placement of belt	
	cECG	Teichmann et al., 2012	0.10–0.85	• Electromagnetic waves	−
				• Motion artifacts	
				• Filter errors	
Optical	Photoplethysmography	Selvaraj et al., 2008; Schäfer and Vagedes, 2013	0.11–0.99	• Latency of pulse wave depending on measuring point	o
				• Varying vascular resistance	
				• Skin type	
				• External light sources	
				• Algorithms that analyze pulse wave kinetics	
Inductive	Magnetic induction	Teichmann et al., 2012	n.a.	• Interference with external Sources	−
				• Motion artifacts	
Vibration	Ballistocardiographie	Teichmann et al., 2012	n.a.	• Motion artifacts	−
				• No direct contact	
				• External vibration interference	
Phonocardiographic	Microphone sensors	Torres-Pereira et al., 1997	n.a.	• Interference with external noises	−
				• Placement of sensors	
Sphygmomanometrical	Blood pressure sensors	Kugler et al., 1997	n.a.	• Motion artifacts	−
				• Contraction of muscles	
				• Incorrect placement of cuffs	

*−, not feasible; o, limited feasibility; +, feasible; n.a., no data available for exercise*.

The gold standard technique for measuring HR is by quantifying the changes of potentials that are caused by the excitation conduction along a myocardial pathway. This conduction produces electrical potentials that can be registered on the skin using an electrocardiograph. In general, 12 electrodes are arranged at defined sites on the body. However, the obtained electrocardiogram (ECG) is only an indicator for the process of excitation. It does not provide information about the actual contraction work of the heart. The application procedure is time consuming and complicated. Therefore, complex knowledge about medical procedures and a clinical setting are essential to obtain valid information. An appropriate and reliable integration in wearables is not feasible so far. A more common use of the electrocardiography are HR breast belts, which also register varying electrical potentials. In contrast to the ECG, only two electrodes are used. The belt can be attached to the thorax. The recorded RR intervals are used to calculate HR. Applied correctly, these belts show high correlation of 0.85–0.99 to the ECG (Weippert et al., 2010). As the sensors need direct skin contact, participants might feel discomfort to undress for application. Another approach is the capacitive electrocardiogram (cECG). The electrodes of the cECG do not need any conductive electrical contact with the participant but can cover distances for example through at least two layer of clothes. Thus, they can be placed in chairs, car seats, and bath tubs. Czaplik et al. (2010) obtained high correlations to conventional ECG at rest in supine position. However, the correlation varied between 0.10 and 0.85 depending on the body position, (breathing) movements, type of clothing, and sweat production of the participants. Additionally, the technological challenges are still high due to motion artifacts and possible filter effects (Teichmann et al., 2012). Therefore, cECG sensors are not feasible to be used in wearables for physical training.

All electrocardiographic measurements can show measuring errors caused by electromagnetic waves of electrical devices and potentials that are caused by muscular activity.

Optical sensors also became more and more popular. Whereas transmissive photoplethysmographic imaging is widely used in clinical settings, reflective photoplethysmography imaging is already applied in smartwatches or activity trackers. Both technologies use a light source and a detector. In transmissive photoplethysmographic sensors, the light source is placed toward the detector, whereas light source and detector are placed on one side of the captured area in reflective photoplethysmography imaging. While the pulse wave is running through the captured area, the amount of arterial blood is slightly increased. The red blood cells absorb the red light leading to different reflections that can be detected. The registered pulse wave therefore represents HR. Although evidence shows a close correspondence of pulse wave and HR (Drinnan et al., 2001; Opalka, 2009), measuring errors can occur due to the latency of the pulse wave and varying vascular resistance (Selvaraj et al., 2008). Therefore, inconsistent findings regarding the reliability can be found depending on location of sensor, experimental condition and performed exercise (0.11–0.99; Schäfer and Vagedes, 2013). Whereas the sensors show high reliability in clinical settings, at rest, and during sleep, the accuracy becomes considerably smaller during movements. Weghorn (2016) found measurements of 118 bpm, while the ECG reference measure was at 65 bpm. Similar results where obtained by Gillinov et al. (2017). Parak and Korhonen (2014) evaluated two photoplethysmographic based HR monitors, where HR measurement lay within a 10 bpm interval in about 87 % of the time compared to the ECG reference heart rate. This incongruence is mainly caused by the signal processing of the pulse wave. In contrast to the sharp increase of the R-spike in the ECG, the pulse wave shows a slow increase and decrease leading to different detection depending on the analyzing algorithm. Additionally, skin color and external light sources might lead to artifacts.

Due to the comfortable handling and application in different locations at the upper and lower extremities, optical sensors have a high potential to be applied in wearables. However, the reliability essentially needs to improve.

Measuring the alternating magnetic field at distinct areas (e.g., wrist) is another measuring approach that has already been implemented in wearable technologies. This technology registers the pulse wave by measuring the regional changes of tissue connectivity and corresponding changes of impedance. It has the advantage that no contact between sensor and measuring site is needed. At rest, the assessment of heart rate variability (HRV) shows very high correlations (0.99–1.00) compared to 3 channel ECG (Kristiansen et al., 2005). However, the interference caused by movements and muscular activity is still very high; reliable values were only achieved under laboratory conditions and at rest (Teichmann et al., 2012). Currently, the technology is not feasible to be used in wearables for physical training.

Infrasonic cardiac vibration sensors (i.e., ballistocardiographic or seismocardiographic sensors) measure the vibration of the human body that is caused by the heart function and the blood flow through the body (Teichmann et al., 2012; Inan et al., 2015). These sensors do not require direct skin contact. Therefore, they can be integrated into devices of daily life (i.e., beds, wheel chair). Shin et al. (2011) obtained a strong correlation (0.97–0.98) on a weighing scale type sensor at rest. However, muscular activity, movements, and floor vibrations may cause measurement errors. Therefore, these sensors do not provide reliable information during physical activity.

Phonocardiographic sensors measure the noise that is produced by the heart function or the blood wave. Modern technology has replaced the stethoscope by a more reliable microphone sensor. However, the reliability of the sensor is not sufficient due to a high amount of interference caused by noise from the environment (Torres-Pereira et al., 1997).

Sphygmographical and sphygmomanometrical sensors measure the differences of blood pressure elicited by systole and diastole. The sphygmo graphical sensor formerly used an inconvenient device attached to the arm, and is therefore not feasible to be used in wearables. Sphygmomanometrical sensors nowadays measure the variance of blood pressure using air pressure cuffs. However, these sensors must be applied by a skilled physician and measurements are non-continuous (Kugler et al., 1997). Therefore, sphygmomanometrical sensors are not feasible for wearables.

Several recent studies showed that accuracy and precision of HR measurement not only depend on the technique of measurement, but is strongly depending on the wearable device used and the completed activity. El-Amrawy and Nounou (2015) compared nine smartwatches and eight fitness trackers. Accuracy for HR measurement (compared to ECG reference heart rate signal) ranged from 92.8 to 99.9 % dependent on the device, and precision ranged from 5.9 to 20.6 %, respectively. Another way to overcome the deficiency of single measurement technologies is to combine sensors obtaining multi-input systems. The developed systems show high reliability and validity (0.993; Brage et al., 2005; Peter et al., 2005).

## 3. Modeling and prediction of heart rate

In the previous section we discussed many difficulties and sources of errors regarding the feasibility of HR measurement approaches for wearables.

While usage of wearables has rapidly increased over the last few years, modeling aspects of health and health care are also helpful in numerous applications as stated in Fone et al. (2003). This is especially accounting for HR. Numerous models have been discussed with regard to HR modeling within the last decades. Physiological models are usually built to simulate a specific behavior of a biological system with high accuracy. These simulations of the human's cardiovascular system encompass a wide range of different purposes and cover wide variations in complexity. For example, Grodins (1959) described the cardiovascular system as “a feedback regulator” and emphasized the importance of identifying the relevant components in a system with inputs and outputs and the connection between both. Therefore, he identified input and output parameters for the right and the left heart, the open pulmonary circuit amongst others, before formalizing and modeling the cardiovascular system. Similar kinds of models on special parts of the cardiovascular system in general can be found in, e.g., Ursino (1998); McLeod (1966); Hotehama et al. (2003); Whittam et al. (1998); Asteroth (2000). A detailed review with focus on the dynamics of the cardiovascular system and physiological models can be found in Lim et al. (2012).

Following a specific purpose, e.g., providing scientific explanations, such physiological “white box” models try to represent special parts of the physiological functions of a human's body. Additionally, there are many techniques which model phenomenological observations. For setting up a phenomenological model, the phenomena have to be defined, which can (or should) be covered by the model. HR response under different load conditions especially in endurance specific context can be described by the following four phenomena:

1. Delayed exponential attenuated HR response to the onset or offset of load, e.g., varying speed, incline or decline conditions on the track (Bunc et al., 1988; Boucsein, 2000);
2. S-shaped HR response with continuous incremental load (Brooke and Hamley, 1972);
3. Cardiac drift during longer activities (Heaps et al., 1994);
4. Exhaustion, which is also defined as “Hitting the wall,” which is described as the moment, where glycogen supplies have been exhausted and energy must be converted from fat (Stevinson and Biddle, 1998).

Additionally, other aspects like a pre-exercise HR or a person's maximum HR can be considered directly or implicitly in a model.

In the remaining, we will focus on phenomenological models because they seem to be more applicable in wearables. Therefore, we will first define different aspects of modeling and differentiate between approximation and prediction. Additionally, we will present different types of models and shortly summarize results of the corresponding studies. This section will end with a discussion of the usage of presented models with regard on modeled physiological phenomena.

### 3.1. Overview of phenomenological models

Phenomenological models and black box models are more applicable than physiological models in terms of approximation and prediction of HR under stress, even if they cannot accurately mirror all effects which occur in a human's body. However, they are used to observe and model essential effects during the training process. Particularly since possibilities of measurement are restricted during training (see 2), an accurate model which depicts too many different physiological aspects is not applicable.

In this paper, we will focus on modeling acute HR responses under stress. As stated in 1, these responses can be subdivided as following: Short-term responses expressed by HR kinetics to the change of load and mid-term responses expressed by individual relationship of stress intensity and HR. These acute responses of human HR under stress are part of numerous phenomenological models.

We can define four different aspects which are relevant when considering HR models from modeling perspective; we have to discriminate between *approximation, short term prediction, session prediction*, and *controlling*, which will be explained in more detail in the following.

As defined in Ludwig et al. (press), many (non-black box) models M can be defined as functions mapping all parameters $\vec{\alpha }$ required by the model, and a stress curve *u*, to an artificially computed HR curve *y*. In this curve both, input (i.e., stress curve) and output (i.e., HR curve), are real time series. The estimated HR at point of time *t* is labeled by *y*(*t*) while $y=M\left(\vec{\alpha },u\right)$, where $\vec{\alpha }\in P$ is the parameter setting^1^ and $u=u_{1},\ldots ,u_{t}\in {\left({\mathbb{R}}^{+}\right)}^{*}$ serves as the model input.

Mathematically, *approximation* is just a curve fitting problem, which is a specific type of optimization problems. The goal of curve fitting is to find the best solution to a specific problem by finding the maximum (or the minimum) of a fitness (or error) function which correlates to the problem. There are several methods for finding local optima—usage of variants of least squares method is most common. In terms of HR modeling, optimization is used to find parameters $\vec{\alpha }$ as optimal as possible, such that the error between the measured HR curve and the modeled HR curve is as small as possible.

Going further, the term *prediction*^2^ can be used to forecast HR, i.e., computing HR values which were not known by the model beforehand and not used for optimizing the model's parameter space. A prediction is dependent on the model parameters previously identified in approximation on different data sets (i.e., approximation is performed on *training data* and prediction on *test data*). In *short term prediction*, we are interested in predicting HR responses to the change of load based on current input data over a certain time horizon. This type of prediction is often used to properly control the stress applied to a subject to prevent unwanted physical effects. If instead the task is to develop a sensitive training plan for a subject over a whole workout session beforehand or to plan a competition, then the input-output relation between imposed stress and resulting HR needs to be predicted over a longer period of time. We use the term *session prediction* to refer to this capability of a model. This means, session prediction is used for predicting a whole time series, such that mid-term HR effects can be modeled as well.

*Controlling* is a special case of HR prediction in this context. It is usually based on short term prediction since the model is used to control the stress which is exposed to a subject, e.g., by an ergometer. Apart from short term prediction, input and output are interchanged in the control application, since the power of an ergometer should be changed dependent on a subject's HR. HR models used for control are often some kind of short term prediction models.

Adjustment of short term prediction models for the usage of session prediction is mathematically possible, but can lead to a lack of accuracy as shown in Ludwig et al. (2015) and Hoffmann and Wiemeyer (2017a). If a short term prediction model makes use of previous HR values, respective previously computed HR values could be used in the corresponding session prediction model. It is possible that the prediction error accumulate quite fast in doing so. Vice versa, models for session prediction can be transformed into models of short term prediction by using the model stepwise.

In general, all HR models have the potential to be used for any application which requires HR modeling with varying accuracy. Some effects might be modeled only indirectly and thus less accurate as in models considering them as phenomena to be modeled directly. Thus phenomenological models cannot represent all possible HR behaviors, but best describe the effects they are built for. For example, Paradiso et al. (2013) stated that they avoid workloads inducing the cardiovascular drift and therefore do not need to include the drift effect in their model. On the other hand, models used for indoor control purposes – like ergometer or treadmill control – do not need to predict future HR values for more than a few seconds.

Table 2 gives an overview of common HR models and summarizes their properties. Each model is first specified by its property of being a black box model, a regression analysis model, or a white box model. Most properties are marked with an “x” if applicable, are further specified, or are marked with “ø” for clarification if a certain property is not specified within the corresponding paper; if the model is used for prediction, the type of prediction is further specified. The number of parameters which need to be optimized is stated where possible; in case of Artificial Neural Networks (ANS), the number results by multiplication of the number of hidden nodes with aggregation of number of input and output nodes (and a bias added if used), since the networks here are built with one hidden layer. Amount of parameters is not specified if a model is not explicitly given and the amount of necessary parameters for optimization is not specified in the correlating paper. The focus for the effects covered by a model is set to the four effects identified as main effects at the beginning of this section—namely delayed exponential attenuated response, S-shaped response, cardiovascular drift, and complete exhaustion. The inclusion of a pre-exercise HR or a person's maximum HR in the model, and the way how stress is included as input is stated here, too. Additionally, some models contain a component for recovery different from the HR response to increasing stress. In this case, the function used for recovery is stated in the table.

**Table 2:** Overview of different specifications of HR models.

**Appr**.	**Paper**	**Specifications**					**Included phenomena**					**Further parameters**		

		**Model type**	**Prediction type**	**Control model**	**Participants**	**Free parameters**	**HR delay**	**S-shape**	**C. Drift**	**Exhaustion**	**Recovery**	**HR**_init_	**HR**_max_	**Stress/Strain**
ANN	Yuchi and Jo, 2008	B	S	ø	1	150	ø	ø	ø	ø	ø	ø	ø	Non-lin.
	Xiao et al., 2011	B	P	ø	ø	ø	ø	ø	ø	ø	ø	ø	ø	Non-lin.
	Mutijarsa et al., 2016	B	S	ø	9	1000	ø	ø	ø	ø	ø	ø	ø	Non-lin.
DE	Cheng et al., 2007	W	S	x	5	5	x	x	ø	ø	tanh	ø	ø	Quad.
	Cheng et al., 2008	W	S	x	6	5	x	x	ø	ø	ø	ø	ø	Quad.
	Paradiso et al., 2013	W	S	x	ø	6	x	x	(x)	ø	ø	ø	ø	Quad.
	Stirling et al., 2008	W	ø	ø	1	4	ø	x	ø	ø	ø	x	x	Polyn.
	Zakynthinaki, 2015	W	ø	ø	2	1	x	x	ø	iAT	iAT	x	x	Polyn.
	Mazzoleni et al., 2016	W	ø	ø	4	14 (11)	ø	x	ø	ø	ø	x	x	Polyn.
Reg.	Hoffmann and Wiemeyer, 2017b	R	ø	ø	4	ø	ø	ø	ø	ø	ø	ø	ø	ø
	Jang et al., 2016	R	ø	ø	217	ø	ø	ø	ø	ø	ø	ø	ø	ø
	Fairbarn et al., 1994	R	ø	ø	462	ø	ø	ø	ø	ø	ø	ø	ø	ø
	Bennett et al., 1993	R	S	ø	9	ø	ø	ø	ø	ø	ø	ø	ø	ø
	Christini et al., 1995	R	ø	ø	9	ø	ø	ø	ø	ø	ø	ø	ø	ø
	Wang et al., 2008	R	ø	ø	10	ø	ø	ø	ø	ø	ø	ø	ø	ø
H/W	Su et al., 2007b	B	S	x	6	ø	ø	ø	ø	ø	ø	ø	ø	ø
	Mohammad et al., 2011	B	S	x	ø	12	ø	ø	ø	ø	ø	ø	ø	ø
	Gonzalez et al., 2016	B	C	ø	5	ø	ø	ø	ø	ø	ø	ø	ø	ø
	Ludwig et al., press	W	C	ø	5	4 (1)	x	x	ø	ø	ø	x	ø	Polyn.
Other	Endler, 2013	W	C	ø	14	4	x	x	x	x	ø	(x)	(x)	Linear
	Dur-e Zehra Baig et al., 2010	W	ø	ø	2	4 · time	ø	ø	ø	ø	ø	x	ø	Linear
	Koenig et al., 2009	W	P	x	10	11 (4)	ø	ø	ø	ø	ø	x	ø	Linear
	Le et al., 2009	W	P	x	ø	4	x	x	x	ø	const.	ø	ø	Linear
	Sinclair et al., 2009	W	C	ø	9	5	x	x	x	ø	x	iAT	ø	Linear
	Yang et al., 2012	R	ø	ø	10	11	ø	x	x	ø	exp.	ø	ø	Linear

*Appr., Approach; ANN, Artificial Neural Network; DE, Differential Equation; Reg., Regression; H/W, Hammerstein-/Wiener-Model; Model Type: B, black box model; R, regression model; W, white box model; Prediction type: S, short term prediction up to 30 s maximum; P, partial training session prediction; C, complete training session prediction; Control Model, Implementation of a controller; Participants, number of participants; Free Parameters, Number of parameters (and after reduction); HR Delay, Delayed exponential attenuated response; S-shape, S-shaped HR response; C. Drift, cardiovascular drift; HR_init_, pre-exercise HR; HR_max_, maximum HR; Stress/Strain, the way how input influences HR; x, applicable; (x), covered indirect; ø, not specified; polyn., polynomial; non-lin., non-linear, quad., quadratic use of the input; iAT, individual aerobic threshold; exp., exponential; const., constant*.

It can clearly be seen that most phenomenological models discussed in this paper are modeled and evaluated for control purposes or for analyzing correlations between HR and specific other measurements or influences. Prediction of complete training sessions beforehand (“C”)—which corresponds to a proper evaluation with a test set independent of training sets used for parameter estimation—is not yet evaluated very well. Regarding the effects, it is noticeable that most models include both, an exponential response to stress and the S-shaped HR response. Many models use some initial or pre-exercise HR, and all other effects are considered more sparsely. Additionally, while only few models incorporate stress linearly, most authors seem to assume a polynomial influence.

Although black box (or gray box) models (e.g., Hammerstein and Wiener models, ANNs) usually do not have physiological correspondence, simulating an existing HR curve or predicting the next few seconds works very well. But when it comes to planning of training or competition, HR approximation of existing training sessions and prediction of only some seconds into the future is not enough any more. For planning a whole training session or simulating a person's capabilities in a competition, HR needs to be predicted over a complete training session. However, black box models tend to overfit in HR response prediction of a complete training session. This is caused by the high number of parameters, which are also often used in non-black-box phenomenological HR models (Ludwig et al., press). Particularly interpretability of a model's parameters is favorable in HR prediction: to model not artifacts but real factors influencing the HR significantly improves the accuracy of prediction. Ludwig et al. (2015) gives a comparison of different types of phenomenological models and presents their accuracy in approximation and prediction of different time horizons of HR. The results illustrate that good accuracy in approximation or prediction of few seconds does not transfer to prediction accuracy in session prediction.

In the following, all considered models are allocated in subsections appropriate to the underlying type of model. Results cited there are always results of approximation (i.e., evaluation of training data set) if not specified otherwise.

#### 3.1.1. Artificial neural networks

Yuchi and Jo (2008) implemented a feedforward ANN to predict HR for the next second based on physical activity (obtained as 3-D acceleration signals), while Mutijarsa et al. (2016) did the same based on cycling cadence. In both networks, the current HR and the respective stress value (physical activity respectively cadence) were used as input variables. HR for the directly following second was set as output. Yuchi and Jo (2008) found a mean absolute error of 3.31 bpm in their test set and found a number of 50 neurons in the hidden layer suitable. Mutijarsa et al. (2016) found a mean absolute error of 3.02 bpm in their test set and identified a number of 333 neurons in the hidden layer via trial and error. The test set is specified as 30 s prediction interval.

Xiao et al. (2009, 2010, 2011) presented different optimization methods based on evolutionary algorithms to train neural networks for HR prediction based on physical activity based on the network described by Yuchi and Jo (2008). HR values were predicted every 30 s for one subject with a short term prediction accuracy of 4.38 bpm (test set) in the mean absolute error.

#### 3.1.2. Differential equation (DE) models

To have a closer look at the differences within the following three DE models, the models share the following general structure:

(1) $$\begin{array}{l} \begin{array}{c} {ẋ}_{1}\left(t\right)=-a_{1}\cdot x_{1}\left(t\right)+a_{2}\cdot x_{2}\left(t\right)+f\left(u\left(t\right)\right)\\ {ẋ}_{2}\left(t\right)=-a_{4}\cdot x_{2}\left(t\right)+g\left(x_{1}\left(t\right),x_{2}\left(t\right)\right)\\ y\left(t\right)=x_{1}\left(t\right) \end{array} \end{array}$$

Here, $a_{i},i\in {\mathbb{N}}^{+}$ are the parameters, *u* serves as model input (stress), and *y* serves as model output (computed HR). The functions *f* and *g* will be specified in the model description to clarify differences in the models.

Cheng et al. (2007) proposed a DE model, which was originally used for treadmill walking and is stated to describe HR behavior during even longer lasting exercises as well as for the recovery phase. One year later, Cheng et al. (2008) published a slightly different DE model used to control speed of a treadmill for regulation of HR in walking at different speeds. In both DE models, the authors formulate two short-term components for different responses in HR changes: One component (*x*_1_) is stated to describe changes in HR based on parasympathetic and sympathetic neural effects as a central response to exercise stress, the second component (*x*_2_) is stated to describe changes in HR based on effects from the hormonal system, increase in body temperature or other slowly-acting effects from the peripheral local metabolism. The output in both models describes the changes in HR from resting HR, while the input signal is set to the walking velocity during the training (and set to 0 for recovery). Velocity is supposed to have a quadratic influence on changes of HR in both models: regarding 1, Cheng et al. (2007) defined:

$$\begin{array}{l} f\left(u\left(t\right)\right)=\frac{a_{2}\cdot u^{2}\left(t\right)}{1+exp\left(-u\left(t\right)+a_{3}\right)},a_{2}=1, \end{array}$$

where the exponential function is used to depict further non-linear effects of the HR; and Cheng et al. (2008) reduced this part of the model to:

$$\begin{array}{l} f\left(u\left(t\right)\right)=a_{2}\cdot u^{2}\left(t\right). \end{array}$$

Furthermore, Cheng et al. (2007) model slow recovery of HR after exercise again with a hyperbolic tangent function within:

$$\begin{array}{l} g\left(x_{1}\left(t\right),x_{2}\left(t\right)\right)=a_{4}\cdot \tanh\left(x_{2}\left(t\right)\right)+a_{5}\cdot x_{1}\left(t\right). \end{array}$$

Only changes of the first component were dependent on input velocity within a sigmoidal function. The five parameters used in this model were estimated using Levenberg-Marquardt. Approximation accuracy is analyzed only visually. The model proposed in Cheng et al. (2008) has no such explicit component to cover slow recovery. While input velocity in this model still only effects changes of the first component, the sigmoidal function here covers changes of the second component, but dependent on the first component, using:

$$\begin{array}{l} g\left(x_{1}\left(t\right),x_{2}\left(t\right)\right)=\frac{a_{4}\cdot x_{1}\left(t\right)}{1+exp\left(-\left(x_{1}\left(t\right)-a_{5}\right)\right)}. \end{array}$$

The possibility to individualize the model using the set of five parameters is retained for this DE model, but the authors estimated fixed parameters based on data of all their subjects to identify a model with no free parameters for their controller design. Approximation accuracy is analyzed only visually, since the focus of the presented work was on controller design and parameter stability. While Scalzi et al. (2012) used the model by Cheng et al. (2008) to describe a new controller design, Paradiso et al. (2013) slightly adapted this model for usage in ergometer cycling. Compared to the original model, they used a new scaling parameter for multiplication with the quadratic input term, i.e.,

$$\begin{array}{l} f\left(u\left(t\right)\right)=a_{6}\cdot u^{2}\left(t\right). \end{array}$$

The authors stated that the model can be used for cycling ergometer control.

A different DE model was proposed by Stirling et al. (2008). Here, changes of HR are modeled as a function dependent on speed (or other intensity measures) and time. Their model is based on two basic components: changes in HR and the exercise demand, which are both dependent on speed and time and constrained by the minimum and maximum HR values of a subject. Three differences are modeled, which are scaled with different parameters and multiplied afterwards: the difference between current HR and minimal HR, between maximum HR and current HR, and between actual exercise demand and current HR. The parameters are used for scaling and to control how quickly HR approaches or diverges from maximum/minimum HR. Parameters do not change during a certain period of training. Changes in parameters over different training seasons are stated to give information about the subject's cardiovascular condition. Approximation accuracy is analyzed only visually. Improved versions of this model with less parameters were presented by Zakynthinaki (2015) and Mazzoleni et al. (2016); we will describe their work in section 3.1.5.

#### 3.1.3. Regression models

Analyzing HR using probabilistic approaches as multiple regression, a frequent goal is to test certain correlations between HR and other parameters^3^. Hoffmann and Wiemeyer (2017b) used multiple regression methods to find factors, which may have a significant effect on changes in HR additional to training effort. They analyzed 19 variables (like restfulness of sleep, nutrition, current mood and others) in terms of their impact on three different parameters of the Bunc equation (Bunc et al., 1988) of HR, i.e., HR at start of the exercise, steady state HR, and a factor used in a basic underlying HR model for slope of the HR curve. The authors found that influences on HR response are very individual, but that physical health, negative mood, the number of intervals in training, as well as time of the day seem to generally influence HR changes. Jang et al. (2016) aimed to find a relationship between running speed and HR using statistical regression methods. In 217 subjects with incremental step tests they analyzed a regression for linear and non-linear HR components; the latter are important because of metabolic demands and cardiac drift effects. In both, inter- and intra-subject analysis, they found a strong correlation between HR and running speed. Smallest errors were achieved with higher regression orders. The regression model of fourth order yielded a correlation of 0.997 and a mean error in HR difference of 2.04 bpm. Similarly, Fairbarn et al. (1994) found linear relationships between HR and oxygen uptake for different aged groups of men and women by analyzing data of 231 subjects during incremental cycle ergometer tests with random effects regression. Richards (1980) provides a good overview comprising (amongst other topics) the HR analysis with statistical measures, multivariate statistical methods, and time series analysis of HR with auto regression. A short workflow of choosing the appropriate statistical method when working with HR data is also given for analysis of raw data.

Bennett et al. (1993) discussed four different autoregressive methods to fit and predict HR time series based on past HR values and noise. They found that the bilinear autoregressive model describes HR dynamics best in comparison to autoregression with and without moving average and polynomial autoregression, but performs poorly in prediction. A similar analysis of Christini et al. (1995) confirms the results. Both concluded that control of HR dynamics should be non-linear.

Wang et al. (2008, 2009) used linear regression and support vector regression (SVR) to examine the relationship between oxygen uptake and other cardiovascular variables like HR. The regression here was focused between oxygen uptake and other cardiovascular factors. Hence, no conclusions were drawn for correlations between HR and other cardiovascular factors. Ludwig et al. (2015) showed that support vector regression can also be used to simulate and predict HR dynamics based upon earlier HR measurements. Esmaeili and Ibeas (2016) applied a particle swarm optimization method for the SVR model proposed by Wang et al. (2008) and claimed to reach better model parameters compared to other studies. Girard et al. (2016) used this model to successfully regulate HR response during treadmill exercise with a PID-controller for treadmill speeds lower than 8 km/h.

#### 3.1.4. Hammerstein and wiener models

Su et al. (2007a,b, 2010) identified a Hammerstein model for HR modeling. Model identification was done separately for the linear and non-linear part of the model by decoupling these parts using pseudorandom binary sequences, which were found to be helpful in this task. Both model parts were identified by machine learning algorithms (e.g., SVR) based on collected experimental treadmill data. The model was used for PID control of the treadmill, which is the focus of the respective work. Based on these Hammerstein model approaches, a modified Hammerstein model is presented and tested by Mohammad et al. (2011). Here, the non-linear part is approximated by a polynomial function.

Gonzalez et al. (2016) focused on approximation and prediction of $\dot{V}O_{2}$ but showed that their identified model can also be applied to HR modeling and prediction. In their work, they analyzed different types of models like autoregressive models with and without a moving average, State-Space models, and Hammerstein-Wiener models and stated that a Hammerstein-Wiener model showed best results in their experiments. Optimization finally leads to a pure Wiener model. In an analysis of five subjects each performing four different bicycle ergometer protocols, average approximation accuracy (training set) of HR was 4.55 bpm, and average session prediction accuracy (test set) was 7.46 bpm.

The model proposed by Ludwig et al. (press) can be illustrated as Wiener model, but has a strong focus on reduction of parameters and thus is presented in section 3.1.5.

#### 3.1.5. Parameter-reduced HR models

Zakynthinaki (2015) stated that HR dynamics in response to movement should be dependent on one parameter describing the cardiovascular condition only. They built their model upon the DE model by Stirling et al. (2008), but added, e.g., different HR phases and time delays and simultaneously reduced parameters to only one global parameter, which represents the cardiovascular condition. The basic structure of their model is still a DE model with difference between current HR and minimal HR, maximum HR, or actual exercise demand. For example, the difference between actual and maximum HR is now part of a sigmoidal function similar to Cheng et al. (2008) instead of scaling this difference by one exponent as before (i.e., ${\left(HR-HR_{max}\right)}^{A}$ with parameter *A*). The number of parameters was reduced via trial-and-error such that all parameters except one could be fixed. The author states that the model is able to predict complete training sessions. The published evaluation is performed visually without numeric values and based on a single protocol for two subjects. In Zakynthinaki (2016), the same model is used to predict different stress courses for synthetic data. Transferability to real training data seems to be not yet proved completely.

Mazzoleni et al. (2016) also built their model based on the DE model by Stirling et al. (2008) for HR modeling in cycling exercises. Additionally, they included a term, which considered torque and cadence, which they stated to be crucial in cycling. They ended up with fourteen parameters, but with a stability analysis using eigenvalues they were able to reduce the number of free parameters to 11 and to restrict ranges of at least two parameters. Parameters were computed based on synthetic data, resulting in a coefficient of determination of *r*^2^ = 0.90, when both cadence and power output are used as model input values.

Koenig et al. (2009) aimed to identify the main effect of change in treadmill speed and human energy expenditure to HR to predict HR during Lokomat walking. Therefore, they calculated the average HR increase for different walking velocities after subtracting a pre-exercise HR value and built a model presented as relay block chart with 11 parameters to scale the effects of the input values including, e.g., fatigue of the subject, and were able to reduce number of free parameters to four.

Ludwig et al. (2016, press) proposed a model which can be described as Wiener model. The basic model has four parameters, which can be reduced to one free parameter. Similar to the idea in Zakynthinaki (2015), this parameter is meant to represent the cardiovascular condition of a person. Furthermore, this model intended to be as simple as possible without lack of accuracy. The model was compared to different other models and yielded lower errors in a complete session prediction. In one study, the average prediction error (test set) was 7.08 bpm in a leave-one-out cross validation of altogether 17 tests of three subjects (Ludwig et al., 2016). In a second study, average approximation error (training set) of 4.95 bpm and an average prediction error (test set) of 7.34 bpm in altogether 20 tests of five subjects was observed (Ludwig et al., press).

#### 3.1.6. Further types of models

Some further model types are occasionally used for HR modeling; to give a short impression of the variety the models will be shortly mentioned in this section.

Dur-e Zehra Baig et al. (2010) compared a linear time invariant (LTI) model with a linear time varying (LTV) model for HR approximation during walking, cycling, and rowing, each at three different intensities, i.e, nine different tasks per subject. The model using parameters varying in time performed better than the LTI model in all analyzed cases with an average mean squared error of 0.158 bpm^2^ for the LTI and 0.071 bpm^2^ for the LTV model over both subjects and all performed tasks.

Le et al. (2009), Sinclair et al. (2009), and Yang et al. (2012) all defined HR as sum of an initial HR value before the start of the exercise and changes due to stress at every point in time. The changes in HR are subdivided into a phase where HR increases, and some phase where the cardiac drift occurs. While Le et al. (2009) differentiated between moderate and exhaustive intensities for the phase of increase, Sinclair et al. (2009) defined a steady-state HR phase including the cardiac drift and used accumulated work instead of plain stress values. Le et al. (2009) and Yang et al. (2012) additionally defined a recovery phase, defined by an exponential function in Yang et al. (2012), and a sum of the HR at anaerobic threshold minus calculated HR values up to exhaustion in Le et al. (2009) – basically the counterpart to their implementation of HR exhaustion. The phase of increase respective HR at moderate intensity is modeled as a single parameter in Sinclair et al. (2009), Le et al. (2009) summed up workload and change in HR at the preceding point in time—each scaled by a parameter—and Yang et al. (2012) additionally added up some noise. The drift is again modeled as a single parameter in Sinclair et al. (2009), while Le et al. (2009) and Yang et al. (2012) used a scaled exponential function depending on the current or last workload respectively.

Endler (2013) adapted a model by Perl (2004) to running, which was initially developed for modeling training processes. PerPot-Run uses speed as input, which is divided antagonistically in a positive and negative potential. The model determines HR as output by flow equations, where positive and negative potentials are effecting the HR with different delays. For prediction usage of the model, it has to be calibrated to an individual subject by a graded incremental test of the subject. PerPot-Run can be used to calculate the individual anaerobic threshold (Endler et al., 2017). Furthermore, it is used to optimize endurance running competitions and training. Endler and Friedrich (2016) presented an extension of PerPot-Run, including incline and decline of tracks.

### 3.2. Usage of HR models and applicability in wearables

A commonly used application for HR models is control of HR on a treadmill (Mazenc et al., 2010; Nguyen et al., 2011; Pătraşcu et al., 2014; Hunt and Fankhauser, 2016; Hunt and Liu, 2017), on a bicycle ergometer (Mohammad et al., 2012; Paradiso et al., 2013; Argha et al., 2014, 2015a,b; Leitner et al., 2014), for gait training (Koenig et al., 2011) or to control strain in exergames (Sinclair et al., 2009). Even apart from strain or stress control, use of HR models is conceivable for many other areas like training planning (Brzostowski et al., 2013; Schäfer et al., 2015), generating individualized training zones based on past training sessions, keeping track of performance development and adjustment of HR training zones, potentially enhancing accuracy by predicting the HR after a model is individualized and adjust the displayed HR according to measurement and model prediction, compensate missing or incorrectly detected HR values [see Jang et al. (2016)], and more.

A simple way to control the individual HR response is by using the closed loop principles of regulatory circuits. Wagner et al. (1993) used the approach of a PD controller for HR control that is solely influenced by the applied load on a bicycle ergometer (*u*). Thus, the load is adapted proportionally and differentially according to the adaptation course of the HR. Since HR response is delayed the load is adapted at distinct time points. The proportional part analyzes the deviation of the desired target (HR_target_) to the actual measured HR (HR_current_). The differential part analyzes the increase of HR represented by the deviation of HR_current_ and the starting HR (HR_start_) within these intervals. The following formula was used:

$$\begin{array}{l} u\left(t\right)=K_{p}\cdot \left({\text{HR}}_{\text{target}}\;-\;{\text{HR}}_{\text{current}}\left(t\right)\right)\\ +\;K_{d}\cdot \left({\text{HR}}_{\text{current}}\left(t\right)\;-\;{\text{HR}}_{\text{start}}\left(t\right)\right) \end{array}$$

Wagner et al. (1993) obtained sufficient results adapting the parameters *K*_*p*_ and *K*_*d*_ individually.

Stirling and Zakynthinaki (2003) provide additional examples how modeling can be used for different processes in sport with a focus on modeling physiological responses to exercise.

In addition to these use cases, applicability of phenomenological models to wearables is an interesting issue. But how can wearables benefit from integration of models? Since several wearables already provide some general training information on a computer based platform, inclusion of HR models could be used to already inform the user during the training about, e.g., the training progress or provide suggestions according to a training plan. Even more, it could help to control the strain a person summons up during a competition [similar to the idea of PerPot-Run by Endler (2013)] by providing useful information about an expected HR or performance progress based on current HR data. Independent of concrete activities or goals, information based on model predictions provided by wearables could help to avoid overstrain, enhance training progresses, and altogether motivate the user to train in an expedient and suitable way. In addition, a well-individualized model could improve the accuracy of wearables by comparing current measurements to predicted HR values.

Some limitations of wearables such as a small screen size and moderate computer performance have to be considered. To provide predictive information during training, it would be necessary that either stress is known beforehand, which might be the case only for very specific applications, or to update the model predictions regularly during the training and based on current strain or stress. Since most HR models use only one input (or input curve), which can be power, velocity, physical activity values, and so forth, the kind of stress considered has to be chosen carefully. For example, in running it might be beneficial to include both, running velocity and slope, which would need to be combined to one stress value for usage in most HR models. While a stress value can be well defined in, e.g., walking, running, and cycling, finding an appropriate measure might be much more difficult in other sports. Here, the use of machine learning algorithms (like ANNs, SVR, or Hammerstein or Wiener models) could be beneficial, since they allow easily to include any desired number of different inputs. However, machine learning algorithms need a huge amount of data to be appropriately trained, and training or updating a model sometimes requires a high computational power and a corresponding computing time depending on the underlying system. Especially for ANNs, a small network with up to 10 neurons should be sufficient for HR prediction. Higher amounts of neurons in the hidden layer can quickly lead to overfitting resulting in bad prediction accuracy. On the other hand, simply using an already trained ANN does not require much time and can easily be executed in real time even on wearables. Therefore, an ANN would be feasible to be used on demand, but should be trained beforehand and not on a wearable.

A potential workability of a model on a wearable is strongly dependent on the specific implementation of this model. Models used for control purposes are often feasible in predicting a few seconds of HR which could also be applicable to wearables. Predicting longer time horizons of HR or controlling a complete training session can also be implemented with models, which are able to accurately predict complete training sessions. Using a suitable implementation, most models will be efficient in just computing current HR values based on a given stress value, while parameter optimization can be time expensive.

In general, individualization of a given model always requires optimization of model parameters, which need data to be trained on and can hardly be performed online during a training. Statistical models and results from statistical analysis can help identifying important parameters affecting HR (like gender, age, body mass index, or similar). With this additional information, HR models could be improved such that less parameters have to be optimized. Adjusting model parameters can certainly be performed faster for less parameters, such that a less complex model with only few parameters could possibly be optimized and adjusted online on a wearable and during training. HR models by Zakynthinaki (2015) and Ludwig et al. (press) are reduced to one parameter and might be good candidates for this purpose. Additionally, results obtained in regression analysis as in Hoffmann and Wiemeyer (2017b) can help reducing necessary parameters in other models. Actual applicability of particular models to wearables has to be analyzed and compared against each other in more detail in the future.

## 4. Summary

Wearables controlling individual strain via HR have the potential to be used as effective and efficient tools for the physical training process. As the HR is integrated in a variety of nested regulatory mechanisms and reflexes, different and highly individual HR kinetics can be observed.

Currently, different sensor technologies measuring HR are available: electrographic sensors, optical sensors, infrasonic vibration sensors, magnetic induction monitoring sensors, phonocardiographic sensors, and sphygmographic sensors. Whereas the electrocardiogram is the “gold standard” for measuring HR, most sensors show high reliability and validity in clinical settings as well. HR breast belts are considered an acceptable compromise of reliability, validity, and usability. Especially optical sensors have a high potential due to high usability and acceptability. However, signal processing, i.e., analysis of pulse wave representing heartbeat, has to be improved. The integration of HR sensors operating on different principles (e.g., photoplethysmography) in wearables for training control is not (yet) feasible due to a variety of possible error sources. Modeling individual responses can be performed using biological and phenomenological models. As biological models are very complex and are more appropriate for offline analysis, they are not feasible to be integrated in wearables for physical training. Phenomenological models in contrast focus specifically on HR response integrating many relevant aspects as cardiac drift or maximum HR. Among other classifications, modeling approaches can be divided into ANN, DE models, regression models, Hammerstein and Wiener models, parameter-reduced HR models, and further models that are occasionally used. The described models can be integrated into wearables for controlling HR on a treadmill, a bike ergometer, for gait training, or strain control within exergames. Additionally, some models can be applied to provide information regarding the long term training process. The feasibility of model implementation in wearables is depending on the reliability of the model, the required processing power, and the output of the model. Currently, pre-trained ANNs, models with individually pre-adapted parameters, or parameter-reduced models seem to be most appropriate for integration into wearables. However, most models were optimized and tested on specific samples. A comparison of the models based on independent data sets is required for objective and reliable evaluation.

## Author contributions

Conceived and designed the manuscript idea: KH and JW; provided substantial contributions to the conception and design of the manuscript, and substantially supported the typesetting: SE; Responsible for sections 1, 2, and 4: KH (writing) and JW (supervising); Responsible for section 3: ML (writing) and AA (supervising). Final supervision of the document: JW. All authors read and approved the final manuscript.

### Conflict of interest statement

The authors declare that the research was conducted in the absence of any commercial or financial relationships that could be construed as a potential conflict of interest.

## Footnotes

^1^Since HR response is delayed, HR increases after a certain time of physical activity and regeneration in relaxation for example are delayed as well. The speed of these adaption processes is highly personalized, and therefore the models should be parametrized for such individual model components.

^2^In estimation theory estimating the value of a function at a given point in time based on the observations made up to this point is denoted as *filtering* rather than *predicting*.

^3^In this specific context, parameters mean measures or effects.
